# Exploratory Study of the Association between the Severity of Idiopathic Intracranial Hypertension and Electroretinogram Photopic Negative Response Amplitude Obtained Using a Handheld Device

**DOI:** 10.3390/life11050437

**Published:** 2021-05-13

**Authors:** Antony Raharja, Shaun M. Leo, Isabelle Chow, Mathura Indusegaran, Christopher J. Hammond, Omar A. Mahroo, Sui H. Wong

**Affiliations:** 1 Medical Eye Unit, St Thomas’ Hospital, London SE1 7EH, UK; antony.raharja@nhs.net; 2 Department of Ophthalmology, Guys & St Thomas’ NHS Foundation Trust, London SE1 7EH, UK; Isabelle.Chow@gstt.nhs.uk (I.C.); Mathura.Indusegaran@gstt.nhs.uk (M.I.); chris.hammond@kcl.ac.uk (C.J.H.); o.mahroo@ucl.ac.uk (O.A.M.); 3 Institute of Ophthalmology, University College London, London EC1V 9EL, UK; shaun.leo@nhs.net; 4 Moorfields Eye Hospital NHS Foundation Trust, London EC1V 2PD, UK; 5 Section of Ophthalmology, King’s College London, London SE1 7EH, UK

**Keywords:** idiopathic intracranial hypertension, retina, electroretinography, retinal ganglion cells, papilledema, pseudotumor cerebri, optic nerve, vision, optical coherence tomography

## Abstract

The photopic negative response (PhNR) is a negative component of the photopic flash electroretinogram that follows the b-wave and is thought to arise from the retinal ganglion cells. Reduction in its amplitude in idiopathic intracranial hypertension (IIH) has been previously documented using formal electroretinography. This study explored the use of a handheld device (RETeval, LKC technologies, Gaithersburg, MD, USA) in 72 IIH patients of varying stages and severity (and seven controls) and investigated associations between PhNR parameters and disease severity. PhNR amplitudes at 72 ms (P_72_) and p-ratio (ratio to b-wave peak value) differed significantly across groups, with a trend towards smaller amplitudes in those with severe IIH, defined as papilloedema with Modified Frisén Scale (MFS) ≥ 3, retinal nerve fibre layer (RNFL) ≥ 150 μm or atrophic papilloedema (*p* = 0.0048 and *p* = 0.018 for P72 and p-ratio, respectively). PhNR parameters did not correlate with MFS, RNFL thickness, standard automated perimetry mean deviation or macular ganglion cell layer volume. This study suggests that PhNR measurement using a handheld device is feasible and could potentially augment the assessment of disease severity in IIH. The clinical utility of PhNR monitoring in IIH patients requires further investigation.

## 1. Introduction

Idiopathic intracranial hypertension (IIH), also known as pseudotumor cerebri (PTC), is a neuro-ophthalmic condition in which raised intracranial pressure causes axoplasmic stasis in retinal ganglion cells (RGCs) by mechanical compression, leading to clinical papilloedema [1]. It predominantly affects obese women of child-bearing age, and its incidence has been significantly increasing in recent years in association with the obesity epidemic [2,3].

Visual impairment is a devastating consequence of untreated IIH; up to 50% of patients experience a degree of visual loss, with blindness affecting 1–10% of cases [4,5]. Thus, protecting vision is a key principle in IIH management [6]. Treatment is aimed at resolving papilloedema and stabilising visual field status. The current standard of visual assessment in IIH relies on perimetric testing, but its reliability may be affected by patient-related factors such as fatigue or poor fixation. Thus, an objective assessment tool could be helpful to improve stratification of IIH patients to identify those at risk of visual loss.

The PhNR is a potential candidate for such a measure, as it derives primarily from retinal ganglion cells and may be sensitive in the detection of early ganglion cell dysfunction [7,8]. The PhNR represents a negative component of the photopic flash electroretinogram (ERG) that follows the b-wave. Previous studies have demonstrated PhNR reduction in optic neuropathies such as primary open-angle glaucoma, optic nerve atrophy secondary to intracranial lesions, optic neuritis and trauma [9,10,11].

A prior study of 10 IIH patients by Moss et al. showed that PhNR amplitudes were significantly smaller in IIH patients than in healthy controls [12]. Another study of 11 IIH patients showed that reductions in PhNR amplitude were significant only if elicited using full-field stimuli, but not focal macular stimuli [13]. Whilst formal electroretinography is not readily available in many clinical settings, a portable device would have the potential advantage of allowing rapid assessments within the clinics in which the patients are already being reviewed. In this study, we recorded PhNRs from a large cohort of IIH patients using a portable device and explored associations with IIH severity.

## 2. Materials and Methods

### 2.1. Study Design and Participants

In this prospective study, patients attending a neuro-ophthalmology service at a single large centre with a suspected or confirmed diagnosis of IIH (between October 2018 and February 2020) were recruited for ERG recordings. We included all IIH patients in the analysis and excluded those with an alternative neuro-ophthalmic diagnosis or poorly obtained ERG traces. ERGs obtained from individuals with no retinal or neuro-ophthalmic pathologies were taken as controls.

### 2.2. Electroretinogram Recordings Using a Handheld Device

After pharmacological mydriasis and exposure to standard room light, a portable ERG device (RETeval system, LKC technologies, Gaithersburg, MD, USA) was used to deliver red flashes (1.0 photopic cd.s/m^2^; 4-millisecond duration; red LED peak wavelength of 621 nm) at 3.4 Hz on a 10 cd.s/m^2^ blue background (blue LED peak wavelength, 470 nm). Averages were taken from up to 400 flash presentations (delivered in two sets of 200 flashes). ERGs were recorded using a conductive fibre electrode placed in the lower conjunctival fornix.

The device has two settings for most protocols: one (the “Td setting”) is designed for use with natural pupils, in which pupil size is measured continually and stimulus strength is adjusted to achieve the desired retinal illuminance (usually equivalent to that of a standard stimulus delivered through a dilated pupil); the other setting (the “cd setting”) is designed for use with pharmacologically dilated pupils, in which a fixed stimulus strength is delivered, as the pupil diameter is not expected to vary during the test. In our study, patients had undergone pharmacological mydriasis, so the second setting (fixed stimulus strength) was employed.

**Figure 1:**
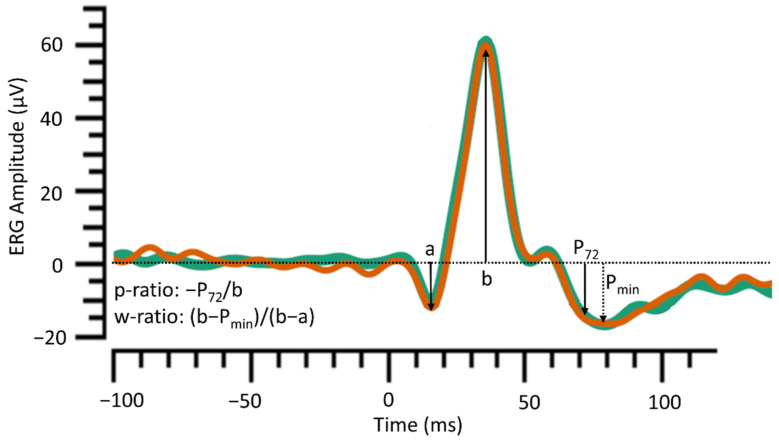
Two ERG traces obtained using a handheld device (RETeval, LKC technologies). Each trace is derived from the average of up to 200 flash presentations. The green trace is the response to the first set of flashes; the orange trace is the response to the second. Two sets of flashes were delivered to check for intrasession reproducibility. The device’s software measures a-wave from the pre-stimulus baseline and b-wave from its peak to the trough of the a-wave (not shown in figure). The PhNR amplitude at 72 ms (P_72_) and minimum PhNR amplitude (P_min_) are measured from baseline. The software calculates p-ratio and w-ratio using b-wave values measured from baseline.

The device’s automated software reports a-wave and b-wave amplitudes and peak times and the following PhNR-related parameters: PhNR amplitudes at 72 ms (P_72_) and at minimum trough after b-wave (P_min_), p-ratio and w-ratio (Figure 1) [11,14]. The a-wave originates from hyperpolarising currents in cone photoreceptors and cone-driven OFF bipolar cells. The b-wave is largely from cone-driven ON bipolar cells but is also shaped by the hyperpolarisation and recovery of OFF bipolar cells [15]. The PhNR is a slow negative component that follows the b-wave and has been shown to arise from retinal ganglion cells [7].

ERG traces were assessed for drift, noise and reproducibility (at least two sets of 200 flashes were delivered in each eye, with the two averaged waveforms compared). The eye with better-quality ERG traces was used for PhNR analysis; if ERG traces from both eyes were rated as good, the eye with poorer visual field function was chosen for analysis (Figure S1A–D). The same principle was applied if one patient had recordings from different ERG visits; the visit with the better-quality recordings was chosen. PhNR amplitudes at minimum trough occurring after 85 ms were excluded from the analysis, as these automatically generated values are potentially spurious (Figure S2).

### 2.3. Clinical Characteristics and Other Parameters of Visual Structure and Function

Case notes were reviewed for demographics (age, sex, ethnicity), anthropometrics (weight, body mass index), clinical characteristics, parameters of visual structure and function (field and optic disc morphology) and cerebrospinal fluid examination (opening pressure and composition analysis).

The severity of optic disc oedema was assessed qualitatively and quantitatively. Colour fundus photographs obtained using a fundus camera (Kowa, VK-2, Tokyo, Japan) were independently graded by two assessors (A.R. and S.H.W.) according to the Modified Frisén Scale (MFS); any disagreement was resolved through discussion [16]. Optic disc atrophy secondary to previous papilloedema (atrophic papilloedema) is assigned grade 6 in our study to allow for analysis of four subjects in our cohort with advanced IIH and pale optic discs [17]. Peripapillary retinal nerve fibre layer (RNFL) thickness and macular ganglion cell layer (mGCL) volume were measured using spectral-domain optical coherence tomography SD-OCT (Heidelberg Engineering, Heidelberg, Germany) and the device’s own automated segmentation. Segmentation was manually checked for errors, but this did not result in any change in the mGCL volume values obtained by automated segmentation. The mGCL volume within the 3.45-millimeter-diameter circle from the fovea was used for analysis. Standard automated perimetry mean deviation (SAP-MD) was obtained using a Humphrey Visual Field Analyzer with 24-2 SITA Standard (Carl Zeiss Meditec, Jena, Germany).

To reflect real-world clinical practice, whereby treatment is primarily aimed at resolving optic disc oedema, IIH patients were grouped according to papilloedema severity: mild (MFS 1-2), severe (MFS ≥ 3, RNFL thickness ≥ 150 μM or atrophic papilloedema) or in remission (papilloedema resolved without atrophy). Both RNFL thickness and MFS were utilised as RNFL thickness alone may be less reliable in higher grades of optic disc swelling [16]. We then explored the correlation between PhNR parameters and visual parameters (papilloedema severity, SAP-MD and current and subsequent mGCL volume loss), and clinical parameters (BMI, lumbar puncture opening pressure and duration of symptoms).

### 2.4. Statistical Analysis

Data are reported as median (interquartile range, IQR). The distribution of the data was tested using the Kolmogorov–Smirnov test. The Mann–Whitney U test or Fisher’s exact test was used to compare two groups, as appropriate. One-way ANOVA or the Kruskal–Wallis test was used to compare more than two groups, as appropriate. To adjust for multiple comparisons, Bonferroni’s or Dunn’s post hoc test was performed if one-way ANOVA or Kruskal–Wallis yielded a significant p-value. Spearman’s correlation coefficient was calculated to determine the degree of correlation as the data were not normally distributed. All statistical tests were carried out using GraphPad Prism, version 9.0.2 (GraphPad software, San Diego, CA, USA).

## 3. Results

### 3.1. Participant Characteristics

Of the 94 participants, 79 (72 IIH; 7 controls) were included and 15 were excluded due to unreliable ERG recordings taken from both eyes (Figure S1E,F). Of the 72 IIH patients included, 90% were female, 44% were of white ethnicity, the median BMI was 34.7 (30.9–39.5) kg/m^2^ and the median CSF opening pressure was 32 (28–39) cmCSF.

Sixty-two (86%) patients met the Modified Dandy criteria for IIH diagnosis [18]. The remaining patients refused lumbar puncture (six) or had falsely low CSF opening pressure (four) due to treatment, weight loss or CSF leak. All patients received a venogram to exclude cerebral venous sinus thrombosis. Controls attended the clinic due to suspicion of IIH based on incidental neuroimaging findings (four) or anomalous optic discs (three); IIH was excluded based on the absence of symptoms and signs of raised intracranial pressure.

Age was similar between the IIH patients of different disease severity, but the controls were significantly older (Table 1). There were no inter-group differences in BMI, duration of symptoms and mGCL volume. RNFL thickness increased with severity of IIH, but the controls showed a similar RNFL thickness as the IIH (mild) and IIH (remission) groups. Visual loss, assessed by SAP-MD, was greatest in those with severe disease, with no difference between IIH (remission) and IIH (mild) groups. 

### 3.2. Electroretinographic Findings and IIH Severity

PhNR measurement using a handheld ERG is feasible, with 79/94 (84%) participants demonstrating reliable ERG traces from at least one eye. Considering all ERG recordings from both eyes of every participant, 28% were rated unreliable due to a combination of drift (36%) and non-reproducibility of traces (34%).

**Figure 2:**
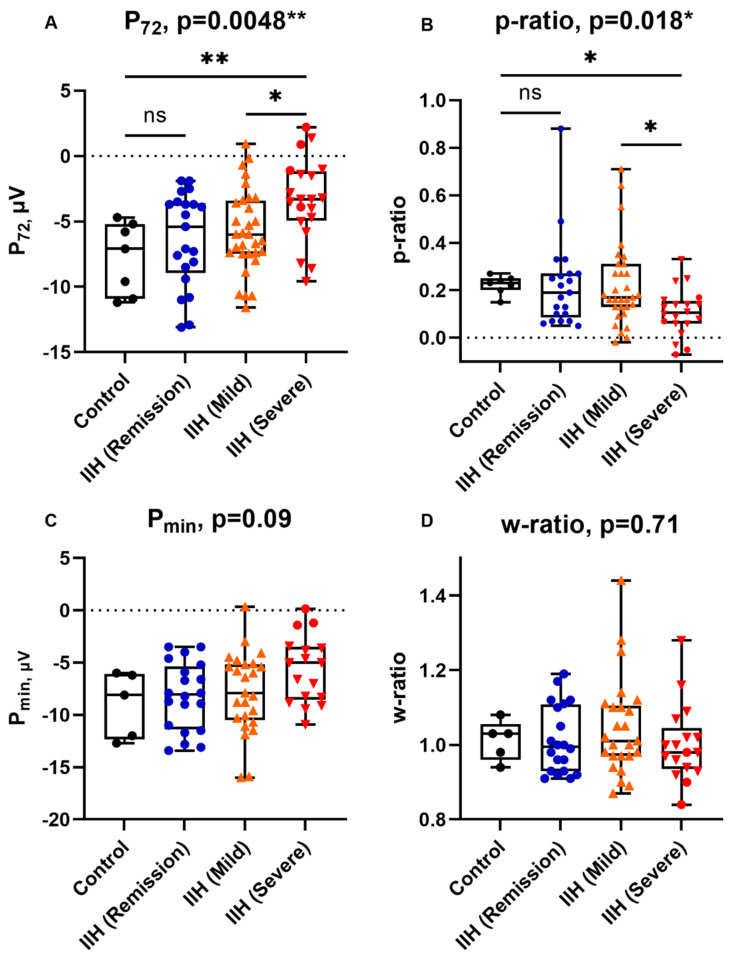
PhNR parameters in different groups: P_72_ (**A**); p-ratio (**B**); P_min_ (**C**); w-ratio (**D**). Points are a dif-ferent colour for each group for clarity. The *p*-values shown in each panel were determined using one-way ANOVA or Kruskal–Wallis test, as appropriate. Pairwise comparisons shown were calculated using follow-up Bonferroni’s and Dunn’s tests for P_72_ and p-ratio, respectively; ns: not significant; *: *p*-value ≤0.05; **: *p*-value ≤ 0.01. Within the IIH (severe) group, patients with atrophic papilloedema are presented as red circles.

There were no significant differences in the amplitudes and peak times of a- and b-waves between groups (Table 2). P_72_ and p-ratio differed significantly across groups, with a trend towards smaller PhNR amplitudes in the more severe group (one-way ANOVA *p* = 0.0048 and Kruskal–Wallis *p* = 0.018 for P_72_ and p-ratio, respectively (Figure 2)).

Bonferroni’s post hoc test for P_72_ showed significant differences between controls and severe IIH patients (*p* = 0.0076) and between mild and severe IIH groups (*p* = 0.032). Dunn’s post hoc test for p-ratio similarly showed significant differences between controls and severe IIH patients (*p* = 0.032) and between mild and severe IIH groups (*p* = 0.026). P_72_ and p-ratio were not significantly different between controls and IIH patients who were in remission (*p* = 0.94 and *p* = 0.99, respectively)

P_min_ and w-ratio measurements did not differ significantly between groups. Compared to other groups, P_min_ appeared to be smaller in severe IIH patients, but this did not reach statistical significance (*p* = 0.09). The inter-group differences in w-ratio were minimal and non-significant (*p* = 0.71).

### 3.3. Correlation between PhNR and Parameters of Visual Structure and Function

**Figure 3:**
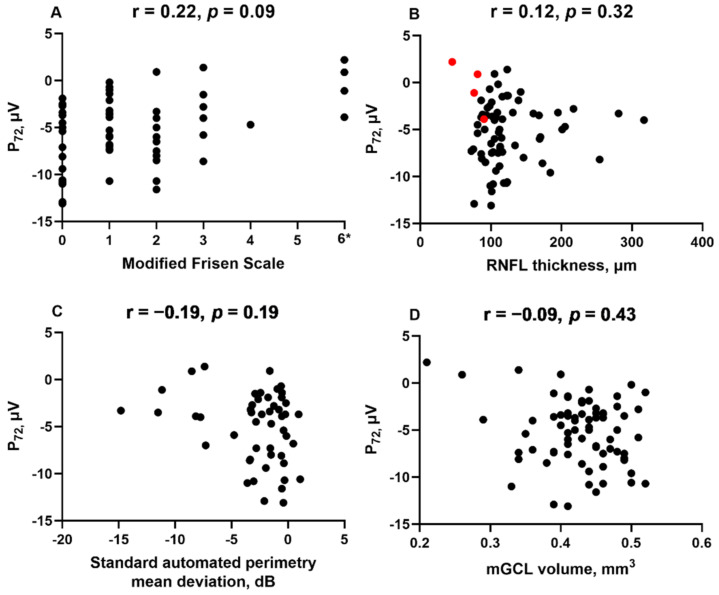
Correlations between P_72_ and visual parameters were assessed using Spearman correlation coefficient. P_72_ does not significantly correlate with the Modified Frisén scale (**A**), peripapillary RNFL thickness (**B**), 24-2 SITA standard Humphrey visual field mean deviation (**C**) or mGCL volume (**D**). * Patients with atrophic papilloedema were assigned a grade of 6 on the Modified Frisén scale and excluded from the RNFL thickness analysis (red circles).

P_72_ did not significantly correlate with MFS (Spearman’s correlation coefficient r = 0.22, *p* = 0.09), peripapillary RNFL thickness (r = 0.12, *p* = 0.32), SAP-MD (r = −0.19, *p* = 0.19) or mGCL volume (r =−0.09, *p* = 0.43). There was a trend towards smaller P_72_ amplitudes in individuals with higher MFS or severe visual field defects (SAP-MD worse than -5dB), but the correlation was not significant (Figure 3). No clear trend was observed between P_72_ and RNFL thickness due to a large spread of data amongst those with minimal to no optic disc swelling. Similar findings were seen for p-ratio, P_min_ and w-ratio.

We then examined if PhNR parameters may predict subsequent neuronal loss. Of the 51 patients with mild or severe IIH, 39 had another mGCL volume measurement after a median duration of 5 (3–9) months. Of these, 15 (38%) had a very minimal reduction in mGCL volume by a median of 0.01 (0.01-0.02) mm^3^, translating to a percentage change of 2.5% (2.3–4.3%). The rest did not experience any neuronal loss. PhNR amplitudes (P_72_, P_min_, p-ratio and w-ratio) did not differ between those who subsequently experienced neuronal loss compared with those who did not (Table S1). None of the PhNR parameters (P_72_, p-ratio, P_min_ and w-ratio) correlated with the loss in mGCL volume (*p* = −0.95, *p* = 0.94, *p* = 0.26 and *p* = 0.12, respectively).

### 3.4. Correlation between PhNR and Clinical Characteristics

We found a weak negative correlation between P_72_ and BMI (Spearman’s correlation coefficient r = −0.27, *p* = 0.023), but P_72_ did not correlate with lumbar puncture opening pressure (r = 0.19, *p* = 0.14) or duration of symptoms (r = 0.046, *p* = 0.72) (Figure S3).

## 4. Discussion

To our knowledge, this is the largest study investigating PhNR in IIH patients. Our study showed that PhNR amplitudes are significantly smaller in severe IIH patients compared to controls, indicating that RGCs’ function may be impaired in severe papilloedema or optic atrophy caused by IIH. This is in keeping with previous studies utilising conventional electrodiagnostic techniques [12,13]. Of note, our study is the first to use a handheld ERG device to show a trend of PhNR attenuation with more severe IIH disease.

Our study demonstrated that PhNR measurement using a handheld device is feasible, quick and might be useful in identifying IIH patients with severe papilloedema causing retinal ganglion cell dysfunction. PhNR amplitude measured at a fixed time point (72 ms, P_72_) and p-ratio appeared to associate better with IIH disease severity compared to P_min_ and w-ratio. The main limitation of the handheld ERG is the significant drift affecting the software’s accuracy in quantifying PhNR amplitudes, but the application of baseline detrending software may improve the yield in future studies [19].

It is currently unclear if PhNR amplitude may recover with prompt treatment of IIH papilloedema. In optic neuritis, the PhNR does not recover, despite clinically effective corticosteroid treatment that improves visual function, suggesting irreversible loss of RGC function in these patients [20]. In glaucoma, substantial intraocular pressure reduction was associated with PhNR improvement [21]. In IIH, there is a paucity of data. Moss et al. described a case of PhNR amplitude recovery with papilloedema improvement in one patient with IIH [12]. Our finding that patients in remission (papilloedema resolved without atrophy) displayed PhNR amplitudes comparable to that of controls may support the notion of PhNR reversibility. This warrants further exploration, as it is unclear if this group had previously experienced PhNR amplitude reduction at the time of active disease.

In our study, PhNR parameters did not individually correlate with modified Frisén Scale, RNFL thickness or the degree of visual field loss. SAP-MD was previously shown to correlate with PhNR in a study involving 10 IIH patients with more severe visual field loss [12]. A subsequent study by the same group could not confirm this [13]. The non-significant correlation in our study might be due to our patient cohort having relatively mild visual dysfunction (median SAP-MD of −1.77) at the time of ERG. A previous study in glaucoma cases showed a negative correlation between RNFL thickness and PhNR amplitudes [22]. The lack of correlation in IIH is not surprising. RNFL thickness increases in papilloedema but then declines due to atrophy or treatment. In our cohort, the spread of PhNR amplitudes in patients with low-grade or no papilloedema is large, suggesting a high degree of variability, or that retinal ganglion cell function may remain normal until severe optic disc swelling or onset of atrophy. As such, RNFL thickness in our cohort of patients at different stages in their disease progression does not strictly correlate with PhNR amplitude.

Patients who experienced a subsequent reduction in mGCL volume did not have significantly different PhNR amplitudes. No correlation was found between PhNR parameters and subsequent change in mGCL volume. Taken together, this suggests that PhNR is unlikely to be helpful in our cohort in predicting those who would experience neuronal loss. Nevertheless, this needs further exploration in studies involving patients with more severe disease, as changes in mGCL volume in our study were minimal.

Obesity has previously been associated as a risk factor for IIH disease, with higher BMI associated with more severe disease [23]. Therefore, the negative correlation between BMI and PhNR in our study needs to be interpreted with caution, as this may be confounded by treatment. A subset analysis excluding those in remission makes this correlation non-significant.

A limitation of the study is the small number of controls and that the controls were significantly older than the IIH patients. Although PhNR amplitudes were not shown to significantly correlate with age in previous studies, trends of lower amplitudes in older individuals have been observed [9,24]. If PhNR amplitudes were to decrease with age (as occurs for a number of ERG components), older controls would be expected to have smaller PhNR amplitudes than the younger IIH patients. However, our study showed that IIH patients have smaller PhNR amplitudes, likely reflecting the effect of the disease on PhNR amplitudes. A comparison with age-matched controls might demonstrate greater differences between controls and IIH patients. As such, this limitation should not affect the validity of the findings of this study.

Another limitation is the relatively low MFS and RNFL thresholds of ≥ 3 and > 150μM, respectively, used to define severe disease in this study. Nevertheless, despite the low cut-offs, this study was able to show statistically significant differences in P_72_ and p-ratio between groups. It is possible that a higher threshold to define severe disease may further augment the between-group differences.

The utility of PhNR monitoring in IIH patients by using a handheld device requires further investigation. There is a considerable overlap in PhNR amplitudes between IIH groups of different severity and within each MFS grade. In addition, given that the IIH (mild) group showed non-significant differences versus controls or the IIH (remission) group, retinal ganglion cell dysfunction may not be appreciated unless papilloedema is severe or optic atrophy has developed. Further studies are required to determine the optimal cut-off for PhNR amplitude, if one exists, to identify those who are at greatest risk of visual deterioration.

## 5. Conclusions

In summary, we have shown an association between some PhNR amplitudes and severity of papilloedema in a large IIH cohort. Future studies are required to further investigate the potential utility of the PhNR as well as the handheld ERG device for disease monitoring in a clinical setting.

## Figures and Tables

**Table 1:** Participant characteristics. Idiopathic intracranial hypertension patients were categorised into groups according to the severity of papilloedema. Remission: papilloedema resolved without atrophy. Mild: Modified Frisén Scale (MFS) 1–2 and RNFL thickness < 150 μm. Severe: MFS ≥ 3, RNFL thickness ≥ 150 μm or atrophic papilloedema.

	Control, *n* = 7	IIH, *n* = 72			*p*-Value
		Remission, *n* = 21	Mild, *n* = 31	Severe, *n* = 20	
White	2/5 (40)	11/20 (55)	7/21 (33)	7/16 (44)	0.99
Female	6/7 (86)	20/21 (95)	28/31(90)	17/20 (85)	0.54
Age, years	53.0 (32.0–65.0)	36.0 (28.5–55.5)	33.0 (26.0–38.0)	30.0 (27.0–37.8)	0.036
Body mass index, Kg/m^2^	34.5 (32.3–42.2)	35.3 (32.0–41.9)	34.2 (31.3–39.3)	33.2 (28.2–40.0)	0.68
Duration of symptoms, months	N/A	28 (12–60)	18 (5.3–36)	24 (7.0–78)	0.58
SAP-MD, db	NA	−1.8 (−3.1, −0.41)	−1.2 (−2.6, −0.34)	−5.4 (−9.2, −1.4)	0.0038
−2 ≤ SAP-MD < −5	NA	8/19 (42)	4/16 (25)	3/14 (21)	
SAP-MD ≤ −5	NA	0/19 (0)	1/16 (6.3)	7/14 (50)	
RNFL thickness, μm	99 (91, 114)	94 (84, 103)	111 (102, 119)	179 (147, 214)	< 0.0001
MFS 1–2	0/7 (0)	0/19 (0)	25/25 (100)	2/16 (12.5)	
MFS ≥ 3 or atrophic papilloedema	0/7 (0)	0/19 (0)	0/25 (0)	10/16 (62.5)	
Atrophic papilloedema	0/7 (0)	0/19 (0)	0/25 (0)	4/16 (25)	
mGCL volume, mm^3^	0.43 (0.39, 0.45)	0.41 (0.39, 0.44)	0.44 (0.41, 0.47)	0.45 (0.37, 0.49)	0.16
CSF opening pressure, cmCSF	NA	30 (25, 36)	33 (28, 40)	33 (29, 40)	0.23

Data are presented as an absolute number (percentage) or median (interquartile range). *p*-values were determined using the Kruskal–Wallis test. *p*-values for sex and age were calculated using Fisher’s exact test, comparing controls with all IIH patients. CSF: cerebrospinal fluid; SAP-MD: standard automated perimetry mean deviation; mGCL: macular ganglion cell layer; MFS: Modified Frisén Scale; RNFL: peripapillary retinal nerve fibre layer.

**Table 2:** Electroretinographic findings.

	Control, *n* = 7	IIH, *n* = 72			*p*-Value
		Remission, *n* = 21	Mild, *n* = 31	Severe, *n* = 20	
**Photopic flash a-wave**					
**Amplitude (μV)**	−8.3 (−11.1, −4.8)	−8.4 (−10.2, 6.3)	−6.8 (−8.4, −4.7)	−5.8 (−8.4, −4.2)	0.070
**Peak time (ms)**	13.5 (13.2, 14.0)	13.2 (13.1, 14.0)	13.3 (12.5, 13.7)	13.2 (12.7, 13.7)	0.53
**Photopic flash b-wave**					
**Amplitude (μV)**	40.5 (33.7, 52.7)	40.2 (33.7, 52.3)	38.8 (30.2, 45.9)	36.5 (26.7, 49.8)	0.77
**Peak time (ms)**	30.4 (29.9, 31.0)	29.7 (27.9, 31.4)	29.2 (27.8, 29.9)	29.2 (28.3, 30.0)	0.29
**PhNR**					
**Amplitude_,_ at 72 ms, P_72_ (μV)**	−7.1 (−10.9, −5.2)	−5.4 (−9.0, −3.6)	−6.0 (−7.5, −3.4)	−3.3 (−4.9, −1.2)	0.0048
**p-ratio**	0.23 (0.20, 0.25)	0.19 (0.09, 0.27)	0.17 (0.13, 0.31)	0.11 (0.06, 0.16)	0.018
**Amplitude at trough, P_min_ (μV)**	−8.1 (−12.4, −6.1)	−8.1 (−11.4, −5.4)	−7.9 (−10.5, −5.2)	−5.0 (−8.6, −3.5)	0.09
**w-ratio**	1.03 (0.96, 1.06)	1.00 (0.93, 1.11)	1.01 (0.97, 1.11)	0.98 (0.94, 1.05)	0.71

Data are presented as median (interquartile range). *p*-values were determined using one-way ANOVA or Kruskal–Wallis test.

## Data Availability

Anonymised data presented in this study are available on reasonable request from the corresponding author and approval by Guy’s and St Thomas’ NHS Foundation trust. Data are not publicly available due to parts of the data containing confidential patient information.

## Supplementary Materials

The following are available online at https://www.mdpi.com/article/10.3390/life11050437/s1, Figure S1: Examples of ERG traces taken from three patients using RETeval handheld ERG device, selected to illustrate drift in some recordings; Figure S2: An example of ERG trace in which Pmin calculation by device software may not be reliable if it occurs after 85 ms; Figure S3: Correlations between P72 and body mass index (A), Lumbar puncture opening pressure (B), and duration of symptoms (C) in IIH patients. Table S1: Comparison of PhNR parameters in those with and without loss in mGCL volume amongst 39 patients with mild or severe IIH who had subsequent mGCL volume measurement.
