# Supplementary material for: Exploratory Study of the Association between the Severity of Idiopathic Intracranial Hypertension and Electroretinogram Photopic Negative Response Amplitude Obtained Using a Handheld Device
Source: Life (Basel). 2021 May 13;11(5):437. doi: 10.3390/life11050437 (PMC8153331; doi:10.3390/life11050437)
Supplement: Supplementary file 1 [file life-11-00437-s001.zip › life-1209239-supplementary.pdf]

life

Supplementary Materials:

Exploratory Study of the Association Between the Severity of Idiopathic Intracranial Hypertension and Electroretinogram Photopic Negative Response Amplitude Obtained Using a Handheld Device

Antony Raharja [1,2], Shaun M. Leo [3,4], Isabelle Chow [2,5], Mathura Indusegaran [2,5], Christopher J. Hammond [2,5], Omar A. Mahroo [2,3,4,5], Sui H. Wong [1,2,4,*]

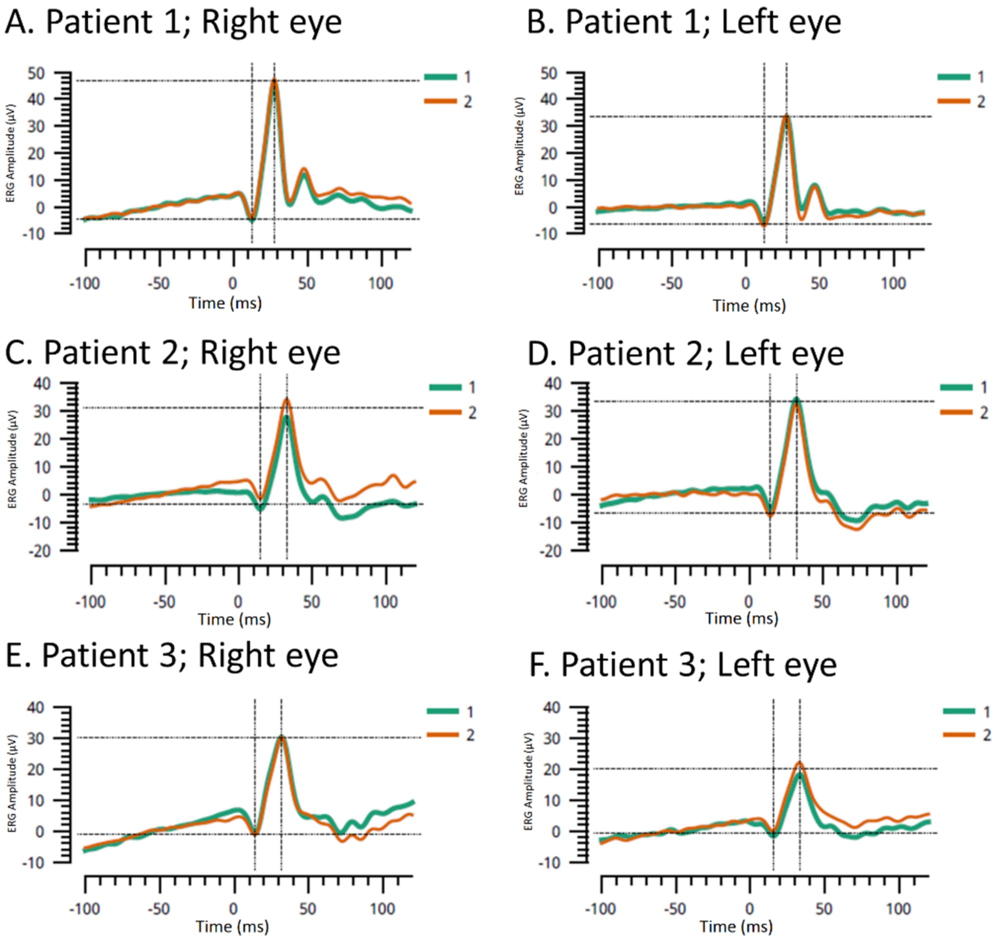

Figure S1. Examples of ERG traces taken from three patients using RETeval handheld ERG device, selected to illustrate drift in some recordings. When assessing ERG traces, we consider drift, reproducibility, noise and blink. The ERG traces from Patient 1 and 2 right eyes (**A and C**) were affected by drift, especially trace 2 from patient 2 right eye. Thus, ERG traces from patient 1 and 2 left eyes (**B and D**) were chosen for PhNR analysis. ERG traces taken from patient 3 both eyes (**E and F**) were not reliable, as there were significant updrift. Therefore, patient 3 was excluded from analysis.

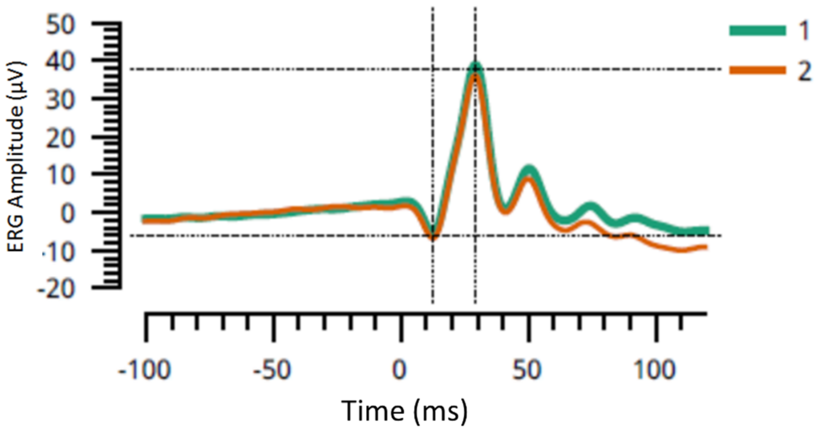

Figure S2. An example of ERG trace in which P_{min} calculation by device software may not be reliable if it occurs after 85ms. In this patient, the device software determined P_{min} to be -9.1μV occurring at 100ms. This is unlikely to accurately represent the true PhNR amplitude in this patient. Therefore, we exclude P_{min} (and w-ratio) from analysis if P_{min} occurs after 85ms.

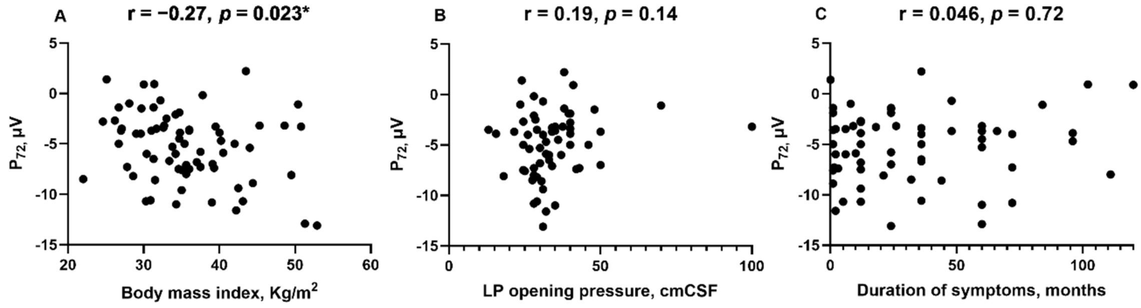

Figure S3. Correlations between P_{72} and body mass index (**A**), Lumbar puncture opening pressure (**B**), and duration of symptoms (**C**) in IIH patients. Spearman correlation coefficient (r) was calculated to determine the degree of correlation.

life

MDPI

Table S1: Comparison of PhNR parameters in those with and without loss in mGCL volume amongst 39 patients with mild or severe IIH who had subsequent mGCL volume measurement.

	Neuronal loss (n = 15)	No Neuronal loss (n = 24)	p-value
**Absolute change in mGCL volume, mm³**	−0.01 (−0.02, −0.01)	0 (0, 0)	**<0.0001**
**Percentage change in mGCL volume, %**	−2.5 (−4.3, −2.3)	0 (0, 0)	
**PhNR amplitude at 72ms, P_{72} (μV)**	−5.9 (−7.3, −2.8)	−5.6 (−7.9, −3.3)	0.64
**p–ratio**	0.14 (0.08, 0.27)	0.16 (0.13, 0.21)	0.49
**PhNR amplitude at trough, P_{min} (μV)**	−8.8 (−10.2, −6.0)	−6.4 (−9.4, −4.9)	0.64
**w–ratio**	1.02 (0.99, 1.21)	1.0 (0.97, 1.08)	0.06

Data variables are expressed as median (interquartile range). P–value was calculated using unpaired t–test or Mann–Whitney U test, as appropriate. PhNR: Photopic negative response; mGCL: macular ganglion cell layer.
